# Beyond the 6-week check-up: exploring the use of physical activity to improve depressive symptoms in women after perinatal loss

**DOI:** 10.1186/1471-2393-15-S1-A18

**Published:** 2015-04-15

**Authors:** Jennifer Huberty

**Affiliations:** 1 Arizona State University, School of Nutrition & Health Promotion, Phoenix, Arizona, USA

## 

The labor, birth, and postpartum periods of women who experience stillbirth are physically similar to women with live birth; however, the negative effects are significantly greater [1]. Women with stillbirth are at three times the risk of depressive symptoms when compared to women with live birth [2]. Depressive states may contribute to weight retention or gain, increased risk of chronic disease (i.e., heart disease), and poor quality of life, and can negatively impact the health of babies born subsequent to loss [3]. Unfortunately, inter-conception interventions to improve the mental and physical health of women after stillbirth are marginal. Treatment may include psychiatric medications and a referral to loss support groups [1,4]. However, these modalities do not consider the unique mental and physical health needs of bereaved mothers, nor do they take into consideration that a majority of women with stillbirth are pregnant or seeking pregnancy within the first year [6-8], and subsequently desire non-pharmacological interventions to cope with their symptoms.

Little is known about using physical activity as a non-pharmacological intervention to cope with stillbirth, despite its known efficacy in improving depressive symptoms in pregnant and postpartum women [8,9]. Women who are active during and after pregnancy have fewer depressive symptoms and report better mood as compared to inactive pregnant and post-partum women [10,11]. This may also be true for women with stillbirth. In a recent qualitative study [12] women with stillbirth who reported regular physical activity participation experienced mental, emotional, and physical benefits that helped them cope with their grief. Even those that were not regularly active reported that when they were active they felt better, had a better mindset and more energy. In the same study, women with stillbirth reported barriers to physical activity participation different than those typically reported in women with live births. Women attributed their lack of activity to emotional symptoms and diminished motivation, being tired and feeling guilt, having a post-pregnant body with no baby, and being confronted with other babies (i.e., exercise in public settings, outside the home). Understanding specific physical activity preferences for these women could inform targeted inter-conception physical activity interventions.

In another study, 175 women with a stillbirth in the preceding year completed a survey to determine women’s preferences for physical activity after loss [12]. Almost 40% were using activity as a means to cope with depressive symptoms, anxiety, and/or grief associated with the death of their baby. Women with stillbirth reported depressive symptoms, weight loss, and better overall physical health (i.e., fitness) as the most important reasons for participating in physical activity. Most preferred activities for coping included walking, jogging, and yoga. Although less than one fourth of the sample reported using yoga as a means to cope with depressive symptoms, half were interested in using yoga to cope and preferred yoga in their homes.

The aforementioned studies provide information necessary for healthcare providers to target inter-conception interventions to improve the mental and physical health of women with stillbirth. Interventions may include: (1) education from health care providers about the benefits of physical activity, (2) exercise groups that incorporate social support from other women with stillbirth, (3) strategies to help women overcome specific barriers related to physical activity and encourage and guide women to use physical activity to cope with their grief, and (4) home-based interventions that incorporate yoga as a means to cope with depressive symptoms. More research is warranted.
